# Advancements in Dairy Research: Exploring Nutritional Strategies, Enhancing Raw Milk Quality and Innovations—Unveiling the Topic “New Insights into Milk 2.0”

**DOI:** 10.3390/ani14131870

**Published:** 2024-06-25

**Authors:** Hasitha Priyashantha, Janak K. Vidanarachchi

**Affiliations:** 1 Department of Molecular Sciences, Swedish University of Agricultural Sciences, SE-750 07 Uppsala, Sweden; 2 Department of Animal Science, Faculty of Agriculture, University of Peradeniya, Peradeniya 20400, Sri Lanka; janakvid@agri.pdn.ac.lk

Dairy research continues to evolve, exploring innovative approaches in farm animal nutrition, milk composition analysis, disease management and the development of functional dairy products. This editorial synthesizes findings from several studies compiled in the topic “New Insights into Milk 2.0” co-published by *Animals*, *Dairy* and *Foods*, each addressing distinct aspects of dairy science. Within the dynamic landscape of dairy research, a complex interplay of factors defines the foundational and crucial link between nutritional strategies, raw milk quality, composition and the evolution of functional dairy products, as graphically summarized in Figure 1. The pursuit of scientific understanding in this domain has propelled us into an era marked by continuous exploration and discovery, encapsulated in the comprehensive insights provided by the eight research articles included in this topic.

In the dynamic discipline of contemporary dairy science, a meticulous examination of nutritional strategies is crucial. At the heart of these advancements lies the integration of cutting-edge nutritional methodologies, reshaping strategies in sustainable dairy production. Beyond traditional boundaries, our exploration delves into the complexities of milk fatty acid composition, where molecular intricacies shaped by ration types, parity, lactation phase and seasonal variations are meticulously scrutinized. The pervasive challenge of mastitis, a prevalent ailment in the dairy sector, takes a prominent role in our exploration. This not only highlights the scientific rigor applied to disease diagnostics but also underscores the crucial role of microbial considerations in maintaining the health of dairy herds. The exploration of sow colostrum and milk, grounded in a profound understanding of lipid metabolism, carries implications not only for sow nutrition but also for the potential development of specialized milk fat equivalents—a prospect with far-reaching consequences for piglet nutrition and health. Navigating this expansive terrain, the study of fermented goat colostrum emerges as a novel frontier in the area of functional dairy products.

Integral to the aforementioned comprehensive exploration is the integration of predictive modelling through mid-infrared spectrometry, serving as a basis for technological procedure in the study of milk. This emphasizes the indispensable connection between farming practices, milk quality and the broader interconnection of dairy research. Rooted in the crucial link between raw milk and dairy products, this editorial venture is aligned with the essential theme presented in the “Topic Information” section. In addressing the continued pursuit of knowledge and understanding regarding the factors associated with milk and dairy product quality, our collective aim is to present a compendium of high-quality scientific research studies to illuminate the advancements in knowledge concerning raw milk, fostering its suitability for the production of high-quality dairy products. Within this exploration, we celebrate the multifaceted aspects covering milk and resulting product quality, driven by primary production factors. Our commitment extends to unravelling the complex relationship between milk and dairy product quality, thus contributing to the ever-evolving landscape of dairy science. The below subsections provide a short summary of articles included in the aforementioned topic.

This editorial investigates the potential of replacing soybean meal with a mixture of locally produced flaxseed and lupins in the diets of dairy cows as shown by Nanas et al. [1]. The authors aimed to assess this mixture’s impact on milk yield, composition and fertility parameters during the transition period, and the results indicated that the dietary treatment did not affect milk yield or composition but led to earlier postpartum estrus expression and conception in cows. Furthermore, no negative effects on milk quality nor oxidative status of animals were observed, suggesting that this dietary modification could be a profitable and sustainable alternative for dairy farms.

The article by Rodríguez-Bermúdez et al. [2] included in this editorial presents an in-depth analysis of fatty acid composition in cow’s milk, focusing on various factors such as the type of ration, parity, lactation phase and season. The study reveals significant variations in milk fatty acid profiles based on the aforementioned factors, indicating the complexity of milk composition. For instance, milk from cows fed total mixed rations with corn silage showed different fatty acid content compared to milk from cows fed pasture-based diets. Additionally, parity, season and lactation phase were found to significantly influence fatty acid composition. These findings highlight the importance of understanding these factors in relation to milk quality, animal health and consumer preferences, highlighting the need for further research in said area.

This editorial includes a study by Ren et al. [3], who examine the differences between fatty acid composition and triacylglycerol (TAG) in sow milk and piglet formula fats, with this study highlighting the importance of understanding these distinctions for optimal piglet growth. The study analyzed total fatty acid and sn-2 fatty acid compositions, as well as TAG species in sow colostrum, sow milk and piglet formulas. Significant differences were observed in the concentrations of medium-chain saturated fatty acids (MC-SFAs) and distributions of palmitic acid (P) and oleic (O)/linoleic (L) acid. Notably, sow milk fats exhibited a higher proportion of palmitic acid located at the sn-2 position of TAGs compared to piglet formula fats. The findings suggest that the unique triacylglycerol structure of sow milk fats, with a large amount of sn-2-esterified palmitic acid, plays a key role in improving fat and calcium absorption. The study classified sow colostrum, milk and piglet formulas into three distinct groups based on their fatty acid compositions and TAG species, providing valuable insights for the design of sow milk fat equivalents and piglet formulas. It emphasizes the importance of considering TAG species in formulating oils for piglet formulas to ensure optimal nutrition and growth. This article offers valuable insights into milk composition, albeit focusing on sow milk and piglet formulas rather than commonly used commercial dairy products. Despite this difference, the study provides novel perspectives on milk quality by examining fatty acid composition and TAG species. While the subject matter may diverge from mainstream dairy industry practices, its exploration of raw milk characteristics aligns with the broader aim of advancing knowledge on milk quality and its implications.

This editorial addresses the dairy industry’s interest in developing a detection tool for pasture-based milk certify the protected designation of origin for certain dairy products. As grazing influences milk composition, the study by Soyeurt et al. [4] aims to develop a predictive model for milk using mid-infrared (MIR) spectrometry to classify milk based on whether it is produced from a grass-based diet or not. Due to the lack of grazing calendars from farms, the study innovatively uses the standard farming practices in southern Belgium combined with a large-scale milk database containing 48 milk composition-related traits. The models developed achieved around 90% accuracy in distinguishing milk from a supposed grass-based diet. The probability of belonging to the grass class could potentially be used as a tool to confirm the labeling of dairy products based on grazing days. The research highlights the importance of detecting milk from grass-based diets and the innovative approach of using farming practices and indirect indicators like the month of testing. The study’s findings offer insights into different feeding strategies and are recommended for future research on accurately estimating grazing periods for cattle. Ultimately, the long-term objective is to create a tool capable of automatically estimating cattle grazing periods, providing valuable information for the dairy industry.

This editorial highlights the study by Flórez et al. [5] on the importance of rapidly quantifying endotoxins in raw milk samples for differentiating between Gram-positive and Gram-negative mastitis, aiding in their clinical management. Mastitis, a common disease in dairy cattle, results in significant losses in the dairy industry and affects animal welfare. Endotoxins released from bacterial lysis play a crucial role in the clinical course of Gram-negative-associated mastitis, influencing antibiotic treatment decisions. To address this need, the authors validated a kinetic turbidimetric assay based on Limulus amebocyte lysate (LAL) for endotoxin quantification in milk samples. The assay, adapted for milk samples through filtration and dilution, demonstrated robustness and utility in identifying coliform mastitis and quantifying endotoxin activity in bulk and commercial milk samples. While the assay shows promise for diagnosing coliform mastitis, further research is required to assess its efficacy in other Gram-negative-associated mastitis cases and throughout the clinical course of these infections, including post-antibiotic treatment. Overall, the developed assay by Flórez et al. [5] offers a standardized method for detecting endotoxins in raw and commercial cow milk, potentially improving mastitis management in dairy cattle.

In this editorial, Bochniarz et al. [6] explored the link between serum and milk levels of tryptophan (TRP), kynurenine (KYN) and kynurenic acid (KYNA), as well as the activity of indoleamine 2,3-dioxygenase (IDO), in cows with mastitis caused by Prototheca algae. The study aimed to explore the potential of TRP metabolites as markers for Prototheca mastitis, given previous research linking the KYN pathway to bovine mastitis. The research, conducted over two years in Poland, compared samples from healthy cows and those with clinical or subclinical Prototheca mastitis. The findings revealed significantly lower TRP and KYN concentrations in milk from mastitic cows compared to healthy ones, along with elevated IDO activity. Interestingly, TRP and KYN concentrations in serum showed less disparity between the two groups. Hence, this editorial highlights the potential utility of TRP and KYN concentrations, as well as IDO activity, as markers for mastitis caused by infectious agents such as Prototheca spp. These findings contribute to our understanding of Prototheca pathogenesis and may aid in mastitis diagnosis, particularly in cases with subtle symptoms.

An investigation by Johansson et al. [7] into raw milk quality revealed age-related differences in technological properties. This observation was made for older cows, which exhibited higher plasmin activity, while younger cows showed elevated plasminogen-derived activity and total proteolysis. Breed-specific effects were also observed, which emphasizes the importance of considering both age and breed in evaluating raw milk quality. However, in this study, authors found minor differences in the composition and technological properties investigated, except for variations in plasmin and plasminogen-related activities. Hence, the overall impact on milk quality was deemed negligible. Therefore, this editorial emphasizes the feasibility of increasing the lifespan of dairy cows without significant effects on milk quality, provided that somatic cell count levels remain within acceptable ranges. This emphasizes the need for continued research to optimize dairy farming practices while maintaining milk quality standards.

This editorial discusses the study published by González-Navarro et al. [8] on the potential of goat colostrum to produce a yoghurt-type product, exploring its suitability as a novel functional dairy food. Colostrum, the first milk produced by mammals after giving birth, contains higher levels of immunoglobulins, growth factors, hormones and antimicrobial enzymes compared to regular milk, making it a subject of interest for sports supplements or nutraceuticals. Traditionally overlooked in dairy product production, surplus colostrum due to increased artificial lactation in dairy goat production systems presents an opportunity to explore its utilization in fermented milk production. The study evaluated the physicochemical, technological, mechanical, microbiological and sensorial characteristics of goat colostrum yoghurt compared to regular goat milk yoghurt. The findings suggested that fermented colostrum has similar features to regular goat milk yoghurt but with higher protein and fat content, along with improved aroma and consistency. The sensory analysis performed indicated high consumer acceptance of colostrum yoghurt, making it a promising option for diversifying dairy product offers and potentially providing health benefits to consumers. Overall, fermenting goat colostrum with yoghurt-specific cultures presents an interesting alternative for utilizing surplus colostrum from dairy goat farms and offering a novel functional product appreciated by consumers.

In this comprehensive editorial for the topic “New Insights into Milk 2.0,” co-published by *Animals*, *Dairy* and *Foods*, recent developments in dairy research are highlighted, focusing on three crucial areas—nutritional strategies, raw milk quality enhancement, and innovation in functional dairy products. Keywords included in this topical collection are summarized in Figure 2, concluding the areas covered within this editorial, based on the keywords published within the aforementioned topic. The editorial investigates the latest findings and emerging trends shaping dairy cow nutrition, emphasizing strategies to optimize milk production while ensuring animal health and well-being. Additionally, advancements in raw milk quality assessment, including novel approaches to monitor and improve milk composition, safety and freshness throughout the production chain, are examined. Furthermore, this editorial highlights the role of innovation in creating functional dairy products, suggesting a revolutionization of dairy farming practices, from precision farming techniques to the application of artificial intelligence and robotics in herd management and milk processing. These technological advancements enhance efficiency, productivity and sustainability by reducing environmental impact and resource consumption.

Looking ahead, this editorial recommends future prospects and challenges in dairy research, envisioning the development of advanced nutritional interventions tailored to individual cow needs and environmental conditions. It anticipates further innovations in raw milk quality control, such as real-time monitoring systems and predictive analytics, to ensure the delivery of safe and high-quality dairy products to consumers worldwide.

Overall, these diverse studies contribute valuable insights to the dairy industry, ranging from nutritional strategies and milk composition analysis to mastitis management and the development of functional dairy products. We underline the multidimensional nature of dairy research, emphasizing its significance for industry stakeholders and consumers alike. In conclusion, this topic on “New Insights into Milk 2.0” sets the stage for a rigorous exploration of dairy science, marked by scientific rigor, methodological innovation and a commitment to unraveling the complexities defining the interface between nutritional strategies, raw milk quality and the evolution of functional dairy products.

## Figures and Tables

**Figure 1:**
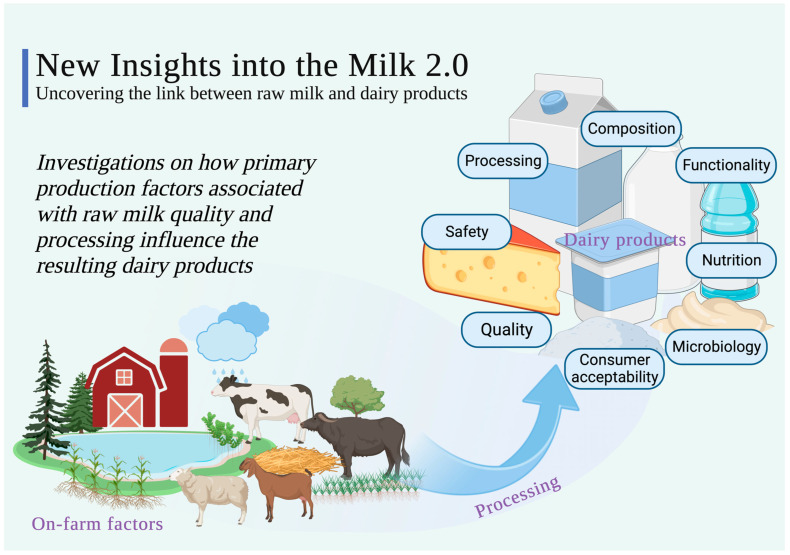
Graphical illustration of areas covered within this topic “New Insights into Milk 2.0” co-published by *Animals*, *Dairy* and *Foods*.

**Figure 2:**
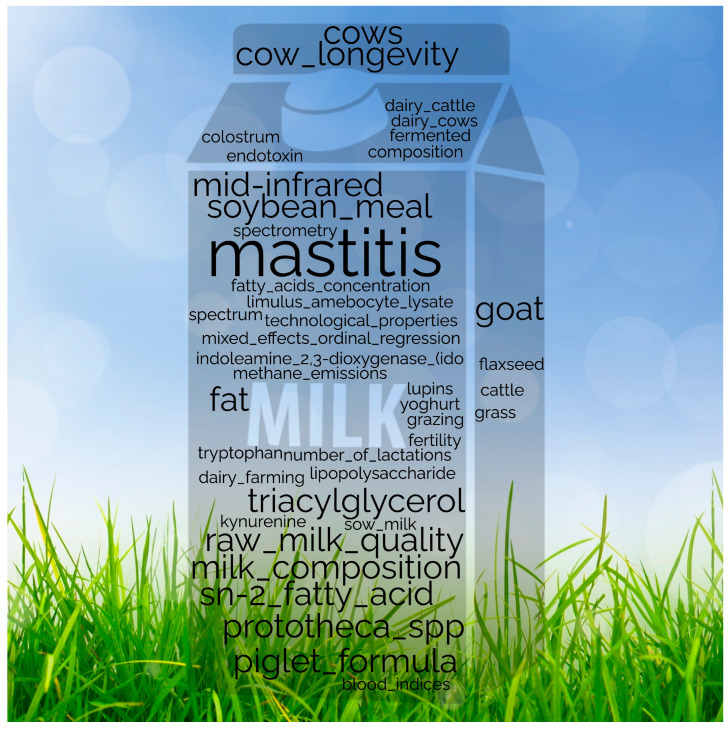
Keywords included in this topic on “New Insights into Milk 2.0.
